# ORION, a new method for root cause analysis of blood and body fluid exposures

**DOI:** 10.1186/2047-2994-4-S1-P266

**Published:** 2015-06-16

**Authors:** C Bervas, S Lafossas, V Fichet, A-G Venier, P Parneix

**Affiliations:** 1 CCLIN Sud-Ouest, France; 2 Centre Hospitalier de Rochefort, France

## Introduction

Blood and Body Fluid Exposures (BFFEs) involve a complex sequence of events combining technical, human and organizational factors. Performing root cause analysis (RCA) of these events is promoted to improve safety. We conducted a RCA of a BBFE in a radiology unit using a recently developed French method called Orion.

## Objectives

The objective was to identify how the BBFE had happened and to implement actions to prevent its reoccurrence.

## Methods

The starting point of the RCA was the report of a BBFE by a radiologist to occupational medicine. BBFE occurred during a non scheduled breast biopsy. Analysis was conducted in collaboration with occupational medicine and the Southwestern Centre for Healthcare Associated Infection Control. ORION comprises six steps: collecting data; rebuilding the chronology; identifying gaps; identifying contributing and influential factors; proposing actions to implement; writing the analysis report.

## Results

The detailed chronology of events before, during and after the BBFE identified many gaps. The main influential factor was a sub-optimal organization during the breast biopsy: no protocol, inadequate room and time slot. Three corrective measures were retained: providing adequate safety container closer to the care procedure; providing adequate medical device to drag the carrot, reorganizing the care with an additionnal microbiopsy session close to RMI session.

## Conclusion

This first use of the ORION method to analyse a BBFE proved successful. This method seems quasi-intuitive and easier to conduct than previously described methods because it relies on a detailed chronology. It allows the implementation of BBFEs preventive measures and promotes collaborative teamwork.

## Disclosure of interest

None declared.

